# Influence of Augmented Visual Feedback on Balance Control in Unilateral Transfemoral Amputees

**DOI:** 10.3389/fnins.2021.727527

**Published:** 2021-09-13

**Authors:** Katharina Fuchs, Thomas Krauskopf, Torben B. Lauck, Lukas Klein, Marc Mueller, Georg W. Herget, Vinzenz Von Tscharner, Norman Stutzig, Thomas Stieglitz, Cristian Pasluosta

**Affiliations:** ^1^Department of Microsystems Engineering, Laboratory for Biomedical Microtechnology, University of Freiburg, Freiburg im Breisgau, Germany; ^2^BrainLinks-BrainTools, University of Freiburg, Freiburg im Breisgau, Germany; ^3^Department of Orthopedics and Trauma Surgery, Medical Center, Faculty of Medicine, University of Freiburg, Freiburg im Breisgau, Germany; ^4^Sanitätshaus Pfänder, Freiburg im Breisgau, Germany; ^5^Human Performance Laboratory, University of Calgary, Calgary, AB, Canada; ^6^Department of Motion and Exercise Science, University of Stuttgart, Stuttgart, Germany; ^7^Bernstein Center Freiburg, University of Freiburg, Freiburg im Breisgau, Germany

**Keywords:** balance control, augmented visual feedback, transfemoral amputee, EnHL, center of pressure

## Abstract

Patients with a lower limb amputation rely more on visual feedback to maintain balance than able-bodied individuals. Altering this sensory modality in amputees thus results in a disrupted postural control. However, little is known about how lower limb amputees cope with augmented visual information during balance tasks. In this study, we investigated how unilateral transfemoral amputees incorporate visual feedback of their center of pressure (CoP) position during quiet standing. Ten transfemoral amputees and ten age-matched able-bodied participants were provided with real-time visual feedback of the position of their CoP while standing on a pressure platform. Their task was to keep their CoP within a small circle in the center of a computer screen placed at eye level, which could be achieved by minimizing their postural sway. The visual feedback was then delayed by 250 and 500 ms and was combined with a two- and five-fold amplification of the CoP displacements. Trials with eyes open without augmented visual feedback as well as with eyes closed were further performed. The overall performance was measured by computing the sway area. We further quantified the dynamics of the CoP adjustments using the entropic half-life (EnHL) to study possible physiological mechanisms behind postural control. Amputees showed an increased sway area compared to the control group. The EnHL values of the amputated leg were significantly higher than those of the intact leg and the dominant and non-dominant leg of controls. This indicates lower dynamics in the CoP adjustments of the amputated leg, which was compensated by increasing the dynamics of the CoP adjustments of the intact leg. Receiving real-time visual feedback of the CoP position did not significantly reduce the sway area neither in amputees nor in controls when comparing with the eyes open condition without visual feedback of the CoP position. Further, with increasing delay and amplification, both groups were able to compensate for small visual perturbations, yet their dynamics were significantly lower when additional information was not received in a physiologically relevant time frame. These findings may be used for future design of neurorehabilitation programs to restore sensory feedback in lower limb amputees.

## Introduction

The loss of a leg is accompanied by adaptations in the postural control system. This implies physical restrictions in everyday life leading to significant psychological burden (Isakov et al., 1992; Nadollek et al., 2002; Ku et al., 2014; Claret et al., 2019). The seemingly simple task of maintaining balance while standing is supported by highly complex sensorimotor processes that are disrupted by the amputation. To maintain an upright posture against the gravitational forces, the center of mass (CoM) needs to be maintained above the base of support (BoS) by adjusting the center of pressure (CoP). To achieve this, sensory stimuli are transmitted to the brain where they are integrated to form an internal representation of the body. This information is transformed into a movement program, which is then transmitted to the muscles and results in muscle activation patterns to counterbalance postural perturbations (Kandel et al., 2013).

Patients with lower limb amputations present a shift in the distribution of their body weight toward the intact limb, leading to an asymmetrical stance (Nadollek et al., 2002; Hlavackova et al., 2011). CoP movement in the anterior-posterior (AP) direction under the intact leg is greater than under the amputated limb. Further, CoP variability increases in the AP direction when vision is removed in patients with unilateral transtibial amputations (Nadollek et al., 2002; Curtze et al., 2012). In the medio lateral (ML) direction, the movement under the intact leg does not differ significantly from the movement of the amputated leg (Nadollek et al., 2002; Hlavackova et al., 2011; Ku et al., 2014). Perturbations in the AP direction are mainly compensated by further use of the ankle strategy. The lack of control of the ankle joint of the prosthetic limb is compensated by an increased ankle movement in the intact leg. Perturbations in the ML direction seem to be successfully compensated by using the hip strategy. Moreover, it has been shown that the mechanical stiffness of the ankle joint of the prosthesis contributes to the control of balance (Curtze et al., 2012).

The visual system provides the central nervous system (CNS) with information from near and far distances, which act as stabilizing clues for the postural system and leads to the perception of self-motion (Wade and Jones, 1997). However, the processing of visual information is not fast enough for the postural system to react to sudden perturbations. The total time to react to a visual stimulus is approximately 150–200 ms (Cameron et al., 2014). The sensory delays of proprioceptive stimuli are estimated to take about 50–60 ms, which is approximately 40–50 ms faster than visual stimuli (Cameron et al., 2014). Further, each sensory modality dominates depending on the postural support and the motor task (Kandel et al., 2013). With sufficient illumination and on stable surfaces, healthy individuals use 70% somatosensory, 20% vestibular, and 10% visual information during upright standing (Peterka, 2002; Horak, 2006). The dependence on vestibular and visual information increases on unstable ground. The ability to re-weight sensory modalities is of great importance to maintain postural control (Peterka, 2002). This ability is severely restricted following an amputation of a lower extremity due to the loss of peripheral somatosensory feedback (Horak, 2006).

Due to the lack of somatosensory and proprioceptive stimuli, visual input thus becomes an important factor in compensating for the postural imbalance caused by the amputation (Barnett et al., 2013). When confronted with visual feedback using a mirror, elder transfemoral amputees were able to integrate enhanced visual biofeedback about their body image to improve the control of upright stance (Hlavackova et al., 2009). The CoP position has been successful employed for augmented visual feedback in balance tasks (Vuillerme et al., 2008; Rougier and Bergeau, 2009; Lakhani and Mansfield, 2015; Kilby et al., 2017; Takeda et al., 2017). However, providing visual feedback of the CoP position does not seem to influence the control of body sway in able-bodied individuals (Danna-Dos-Santos et al., 2008). The effectiveness of visual feedback to improve postural control depends on the approach used to provide visual information (Pinsault and Vuillerme, 2008). For example, providing real-time and amplified (five- and ten-fold) position of the CoP resulted in a reduced CoP excursion in healthy elderly subjects. Yet, delaying visual feedback of the CoP position (250, 500, 750, or 1000 ms) increased the variability of CoP adjustments during quiet standing in the same population (van den Heuvel et al., 2009).

The temporal variation in physiological data is best quantified using measures that evaluate variability at different time scales (Federolf et al., 2015). To quantify the dynamics and structural changes of the CoP adjustments during balance control, time-scale measures such as multi-scale entropy have been proposed (Miller et al., 2006; Stergiou et al., 2006; Ramdani et al., 2009; Hlavackova et al., 2011; Zandiyeh and von Tscharner, 2013; Baltich et al., 2015). This allows the investigation of postural control in relation to mechanical and neurophysiological aspects such as the influence of sensory feedback on movement regulation (Paillard and Noé, 2015). In this sense, the method of the entropic half-life (EnHL) (Zandiyeh and von Tscharner, 2013) has been proposed to evaluate the dynamics of CoP measurements during quiet standing (Zandiyeh and von Tscharner, 2013; Baltich et al., 2015; Pasluosta et al., 2017; Pasluosta et al., 2018; Claret et al., 2019).

Since lower limb amputees rely more on visual feedback, a disruption or alteration of this sensory modality should have a significant impact on their postural control. However, the effect of augmented visual feedback on the performance of static balance tasks in lower limb amputees remains unexplored. The aim of this study was to examine the influence of real-time visual feedback of the CoP location on the postural control of unilateral transfemoral amputees. Visual information of the CoP position was delayed and amplified to quantify the ability to maintain postural stability with disruptive visual feedback. We hypothesized that (1) augmented visual feedback of the CoP position will reduce the sway area in transfemoral amputees and able-bodied controls, (2) increasing delays of augmented visual feedback will increase the sway area in both groups, and (3) augmented visual feedback of the CoP position will affect the dynamics of the CoP adjustments more in the amputee than in the control group.

## Materials and Methods

### Participants

Ten unilateral transfemoral amputees (age: 54,70 ± 13,84 years; height: 175,30 ± 8,90 cm; weight with prosthesis: 77,10 ± 12,60 kg; and years since amputation: 27,40 ± 17,13 years; prosthesis model: 6 with Otto Bock Genium, 2 with Otto Bock C-Leg, 1 with Otto Bock Kenevo knee, and 1 with Otto Bock Genium X3) were recruited in collaboration with Pfänder Orthopedics, Freiburg, Germany. The inclusion criteria were having a unilateral transfemoral amputation without any other orthopedic, neurological, or cardiovascular pre-existing conditions. Ten age-matched (unpaired *t*-test, *t*(9) = 0.00,and*p* =  1.00) able-bodied participants (age: 55,89 ± 5,67 years; height: 173,11 ± 9,14 cm; and weight: 68,89 ± 8,28 kg) were recruited as a control group (Table 1). The able-bodied subjects specified their dominant leg as the propulsive leg with which a step is initiated or the first one used to climb a stair step. All participants had normal or corrected-to-normal vision. All participants signed an informed consent form approved by the ethics committee of the University of Freiburg, Freiburg, Germany (ethical approval N° 230/18).

**TABLE 1:** Mean and standard deviation of the participants characteristics.

**Group**	**Age in years**	**Height in cm**	**Weight in kg**	**Years since amputation**	**Feet width in cm**
All	55.26 ± 10.51	174.26 ± 8.83	73.21 ± 11.14		23.54 ± 4.08
Amputees	54.70 ± 13.84	175.30 ± 8.90	77.10 ± 12.60	27.40 ± 17.13	25.59 ± 3.99
Controls	55.89 ± 5.67	173.11 ± 9.14	68.89 ± 8.28		21.72 ± 3.39

### Experimental Setup and Protocol

**FIGURE 1:**
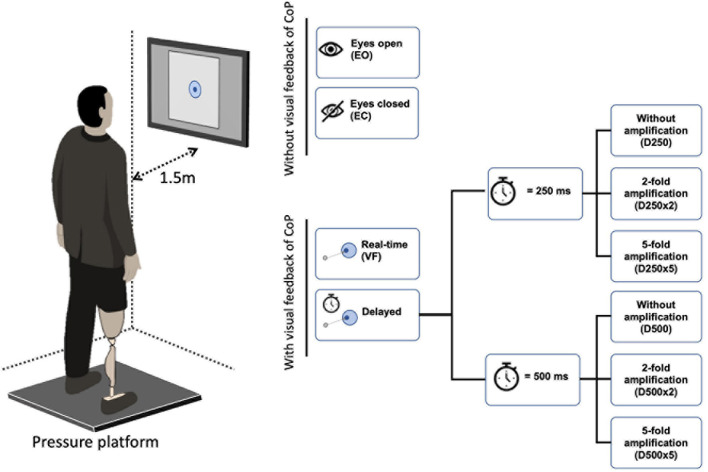
Experimental set up. Participants stood with both legs in an upright position on a pressure platform facing a computer screen, which was located at a distance of 1.50 m from the subject’s body. The feet were placed at shoulder distance in the positive AP direction of the pressure platform. The arms were hanging relaxed on either side of the body. The screen displayed a white field with a light blue concentric circle in the center. The center of pressure (CoP) was shown within the white field as a dark blue dot that moved in relation to the CoP position. The participant’s task was to keep this dark blue dot inside the light blue circle.

Participants stood on an FDM-S pressure platform (zebris Medical GmbH, Isny im Allgäu, Germany) in front of a computer screen located at a distance of 1.50 m away from the subject’s body (Figure 1). The screen showed a white square field (30 × 30 cm) with a light blue concentric circle around its center (target circle) located at eye level. The size of this target circle was calculated from CoP data collected during 30 s of quiet standing with eyes open, gazing at the center of the screen (i.e., at a circle of 50 mm radius). The radius of the target circle was then defined as twice the standard deviation of the CoP data, averaged across direction (i.e., average of the CoP variability in the AP and ML directions).

After the radius of the target circle was defined, real-time CoP measured with the pressure platform (sampled at 100 Hz) was displayed within the white field as a dark blue movable dot with a fixed diameter of 11 mm (Figure 1). The participant’s task was to keep the dark blue circle (i.e., projected CoP position) within the target circle. Three 30-s trials were performed with visual feedback of the CoP position without delay (VF), with a delay of 250 ms (D250) and of 500 ms (D500), with the two-time delays combined with a two- or five-fold amplification of the CoP displacements (D250x2, D250x5, D500x2, D500x5), and with eyes closed (EC). Three 30-sec trials were also performed with eyes open without visual feedback of the CoP position (EO). The conditions were presented in random order. Participants stood with both feet at a shoulder-to-shoulder distance on the pressure platform in an upright position facing the positive AP direction of the pressure platform. Their arms were hanging relaxedly on either side of the body (Figure 1). All participants wore shoes. CoP position under the full body and for each foot was recorded.

### Data Processing

Data processing was performed using Matlab version 2021a (MathWorks, Natick, MA, United States). The CoP data was first bandpass filtered with a wavelet filter (Tscharner and von Schwameder, 2001) with cutoff frequencies of 0.15 and 10 Hz. The first and last 5 s of each measurement were neglected to avoid any transients. The sway area was then calculated as the 95% confidence ellipse area of the CoP data according to the calculations published by Prieto et al. (1996). The sway area values were then averaged across trials to produce one value per condition and per subject. The sway area values of each visual feedback condition (*SA*_*X*_ in Eq. 1) were normalized (*SA*_*Xnorm*_ in Eq. 1) with respect to the values of the EO condition (*SA*_*EO*_ in Eq. 1).


(1) $${\text{SA}}_{X\,n\,o\,r\,m}=\frac{{\text{SA}}_{X}-{\text{SA}}_{E\,O}}{{\text{SA}}_{E\,O}}$$

**FIGURE 2:**
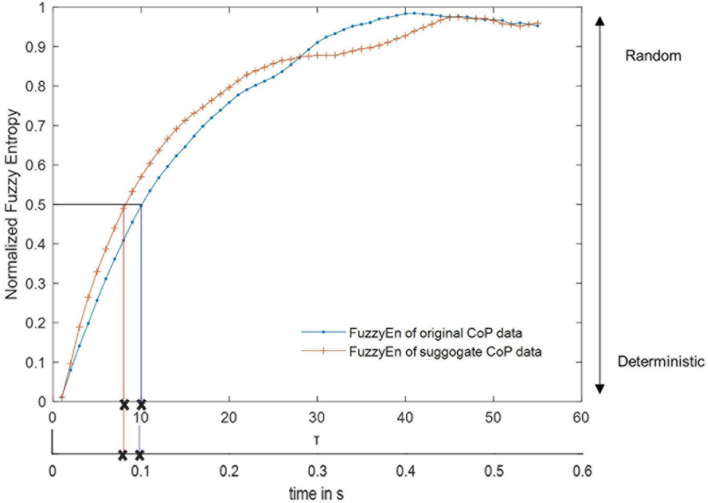
Representative transition curve of the normalized Fuzzy Entropy of the original and surrogate CoP data of an able-bodied control with eyes open, normalized to the Fuzzy Entropy of a random data set with the same length as the original data. The Fuzzy Entropy for every Reshape Scale τ is displayed with a marker. Here, the EnHL of the surrogate data was reached after 8 reshape scales and that of the original data after 10 reshape scales (marked with an **x**). Since the time between each data point is 0.01 s (at a sample frequency of 100 Hz), the EnHL of the surrogate data is 0.08 s, and the EnHL of the original data is 0.1 s.

The dynamics of the CoP adjustments were computed using the EnHL method as published elsewhere (Zandiyeh and von Tscharner, 2013). Briefly, the CoP data were rearranged at different time scales using the reshape scale method (Zandiyeh and von Tscharner, 2013), such that at each reshaping step (in this work, we used 55, corresponding to timescales between 10 and 550 ms) the signal was gradually randomized. After applying each reshape-scales (RS), the fuzzy sample entropy (FuzzyEn, *m* = 3, *r* = 0.7 relative to standard deviation, and expo = 5) was computed on the reshaped signals (Xie et al., 2011). This generated a transition curve of entropy against time scales (Figure 2). The transition curve was normalized with respect to the entropy value of total randomization of the CoP data. The EnHL was then defined as the time scale required to reach half of the maximum entropy (the exact value was calculated by linear interpolation, Figure 2). The EnHL thus represents the time scale at which the CoP signal switches from deterministic to random behavior. In other words, the EnHL represents the time scale at which current CoP adjustments are independent on previous ones.

Center of pressure data are expected to have a deterministic origin and thus it is assumed that the signal contains information over time (Zadeh, 1977). To test this hypothesis, surrogate CoP data of the same length as the original data was computed using the amplitude-adjusted Fourier transform (AAFT) (James et al., 1992). The AAFT preserves the linear autocorrelation of the original signal but randomizes the phase, which destroys the signal information over time. Since the surrogate signals should be more random than the original CoP signals, an analysis with the EnHL method should always yield lower EnHL values for the surrogate signal than for the original signal, ruling out the possibility that the signal originates from random processes.

### Statistical Analysis

The statistical analysis was performed in R (R Core Team, 2021). All data were tested for normal distribution using the Kolmogorov-Smirnov test and Q-Q plots. For the sway area, a two-way mixed ANOVA was performed to test for between-subject factors of group (two levels, controls vs. amputees), and to test for within-subject factor of visual feedback condition (nine levels, EO vs. EC vs. VF vs. D250 vs. D500 vs. D250x2 vs. D250x5 vs. D500x2 vs. D500x5). Subsequent multi-comparison post-hoc pairwise *t*-tests with Bonferroni corrections were performed in case of significant effects in any of the main factors or their interactions.

For the EnHL values, a five-way mixed ANOVA was performed to test for between-subject factors of signal (two levels, original vs. surrogate), group (two levels, control vs. amputee), leg (three levels, intact/dominant vs. prosthetic/non-dominant vs. both), direction (two levels, ML vs. AP), and the within-subject factor of visual feedback condition (nine levels, EO vs. EC vs. VF vs. D250 vs. D500 vs. D250x2 vs. D250x5 vs. D500x2 vs. D500x5).

A four-way mixed ANOVA was then carried out on the EnHL values of the original CoP data to test for between-subject factors of group (two levels, control group vs. amputee), leg (three levels, intact/dominant vs. prosthetic/non-dominant vs. both), direction (two levels, ML vs. AP), and the within-subject factor of visual feedback condition (nine levels, EO vs. EC vs. VF vs. D250 vs. D500 vs. D250x2 vs. D250x5 vs. D500x2 vs. D500x5). In case of significant effects on any of the main factors or interactions, further multi-way ANOVA were performed with subsequent multi-comparison *post-hoc* tests with Bonferroni corrections. In this way, if for example a three-way interaction was significant (e.g., a Group×Direction×Condition effect was significant), a three-way ANOVA for each group was further performed. This process was repeated if subsequent interaction effects were observed.

## Results

### Sway Area

**FIGURE 3:**
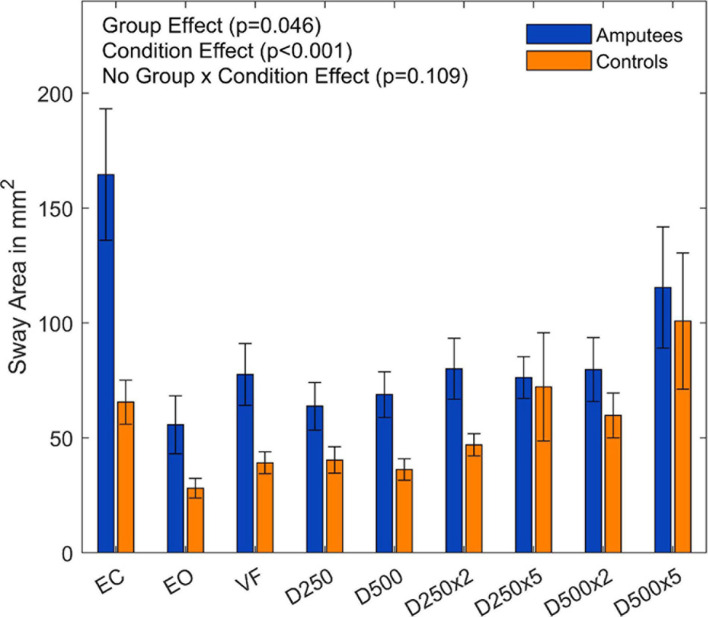
Sway area of amputees and controls for the different augmented visual feedback conditions (VF, visual feedback of CoP position without delay; D250, with a delay of 250 ms; D500, with delay of 500 ms; D250x2, D250x5, D500x2, and D500x5: with delays of 250 and 500 ms combined with a two- or five-fold amplification of the CoP displacements; EC, with eyes closed; and EO, with eyes open without visual feedback of the CoP position). Error bars represent standard errors.

Overall, the sway area of the amputees was significantly higher than that of the control group (F(1,18) = 4.59,p =  0.046, and Supplementary Table 1). There was a significant main effect of visual feedback conditions (F(8,144) = 9.36,p = < 0.001, and Supplementary Table 1). The pairwise *t*-test with Bonferroni correction showed a significant increase for the D250, D250x2, and D500 condition compared to the EC condition (Figure 3 and Table 2). The sway area for the VF condition was significantly higher than the EO condition but lower than the EC condition (Figure 3 and Table 2). The sway area for the D250 condition was significantly lower than for the D500x2 condition (Figure 3 and Table 2).

**TABLE 2:** *p*-values from pairwise *t*-test with Bonferroni-correction to test for significant difference between the sway area data of the different conditions in amputees and controls.

	**D250**	**D250x2**	**D250x5**	**D500**	**D500x2**	**D500x5**	**EC**	**EO**
D250x2	1.000	–	–	–	–	–	–	–
D250x5	1.000	1.000	–	–	–	–	–	–
D500	1.000	0.408	1.000	–	–	–	–	–
D500x2	**0.037**	1.000	1.000	0.306	–	–	–	–
D500x5	0.139	0.464	1.000	0.154	1.000	–	–	–
EC	**0.009**	**0.024**	1.000	**0.009**	0.225	1.000	–	–
EO	0.969	**0.029**	0.807	0.926	**0.002**	**0.042**	**0.002**	–
VF	1.000	1.000	1.000	1.000	1.000	0.517	**0.020**	**0.028**

*Statistically significant values are displayed in bold font (significance level α = 0.05).*

**FIGURE 4:**
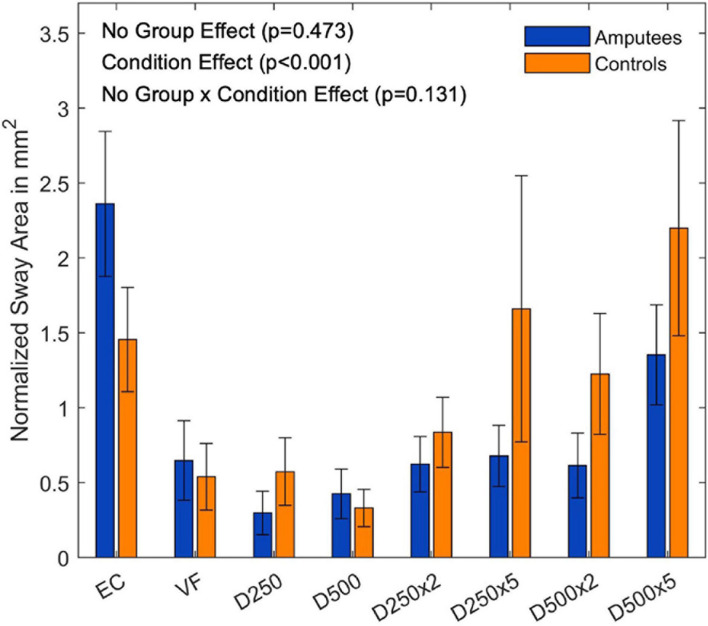
Ratio of the sway area data normalized to the EO condition of each group for the different augmented visual feedback conditions (VF, visual feedback of CoP position without delay; D250, with a delay of 250 ms; D500, with delay of 500 ms; D250x2, D250x5, D500x2, and D500x5: with delays of 250 and 500 ms combined with a two- or five-fold amplification of the CoP displacements; EC, with eyes closed; and EO, with eyes open without visual feedback of the CoP position). Error bars represent standard errors.

There was no group effect for the sway area normalized to the EO condition (F(8,144) = 0.45,p =  0.473, and Supplementary Table 2). There was, however, a significant main effect for the visual conditions (F(8,144) = 7.89,p < 0.001, and Figure 4). The subsequent *t*-tests with Bonferroni correction to compare the visual feedback conditions are listed in the Supplementary Table 3.

### Entropic Half-Life

There was a significant difference between the EnHL values for the five-way ANOVA of the original and the surrogate CoP signals (F(1,216) = 146.91,p < 0.001, and Supplementary Table 4), with the EnHL values of the surrogate data being lower than the EnHL values of the original data.

**FIGURE 5:**
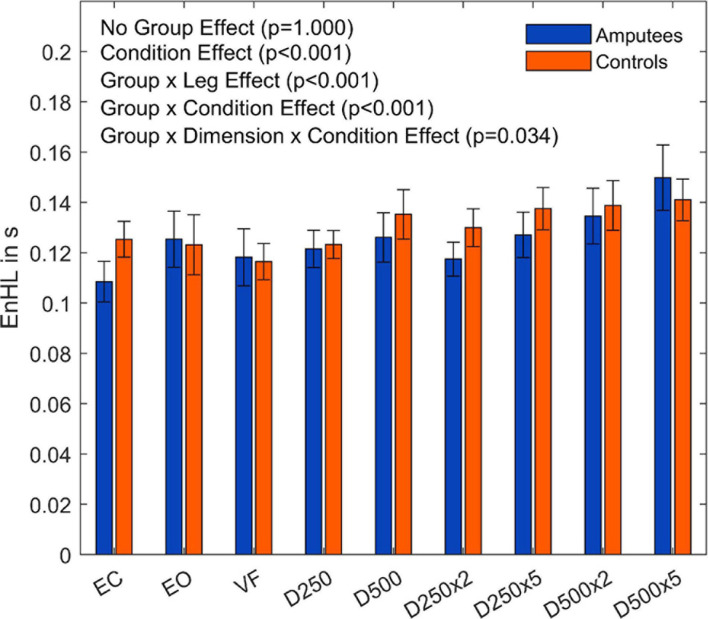
Entropic half-life (EnHL) values of the CoP for both legs of each group for the different augmented visual feedback conditions (VF, visual feedback of CoP position without delay; D250, with a delay of 250 ms; D500, with delay of 500 ms; D250x2, D250x5, D500x2, and D500x5: with delays of 250 and 500 ms combined with a two- or five-fold amplification of the CoP displacements; EC, with eyes closed; and EO, with eyes open without visual feedback of the CoP position). Error bars represent standard errors.

The subsequent four-way ANOVA using the original data showed a main effect for the leg factor (F(2,108) = 21.21,p < 0.001, and Table 3), and the condition factor (F(8,864) =  19.15,p < 0.001, Table 3, and Figure 5). There were interaction effects between group and leg (F(2,108) = 24.06,p < 0.001, Table 3, and Figure 5), between group and condition (F(6,864) = 3.07,p =  0.005, Table 3, and Figure 5) and between group, direction, and condition (F(8,864) = 2.26,p =  0.034, Table 3, and Figure 5). This indicates a significant difference between amputees and controls regarding the dynamics of CoP adjustments with different visual conditions (Table 3).

**TABLE 3:** Four-way mixed ANOVA of the EnHL values from the original signal to test for significant differences between amputees and controls (Group).

**Main- and interaction effects**	***F*-value**	***p*-value**
Group	1.860	0.177
Direction	0.230	0.633
**Leg**	**21.210**	**<0.001**
**Condition**	**19.150**	**<0.001**
Group×Direction	0.590	0.443
**Group×Leg**	**24.060**	**<0.001**
Direction×Leg	0.250	0.778
**Group×Condition**	**3.070**	**0.005**
Direction×Condition	1.150	0.328
Leg×Condition	1.310	0.206
Group×Direction×Leg	0.000	0.996
**Group×Direction×Condition**	**2.260**	**0.034**
Group×Leg×Condition	0.860	0.589
Direction×Leg×Condition	0.860	0.597
Group×Direction×Leg×Condition	0.560	0.881

*Statistically significant values are displayed in bold font.*

There was a significant main effect between the legs of the amputees (F(1,54) = 42.43,p < 0.001, and Supplementary Table 5) but no significant differences were observed between the legs of the able-bodied controls (F(2,54) = 0.28,p <  0.757, and Supplementary Table 6). Both groups showed a significant difference among visual feedback conditions (Amputees: F(8,432) = 13,67,p < 0.001, and Supplementary Table 5; Controls: F(8,432) = 8.83,p < 0.001, and Supplementary Table 6). The results also showed a significant interaction effect between the directions and visual feedback conditions in the group of amputees (F(8,432) = 2.69,p =  0.015, and Supplementary Table 5).

**FIGURE 6:**
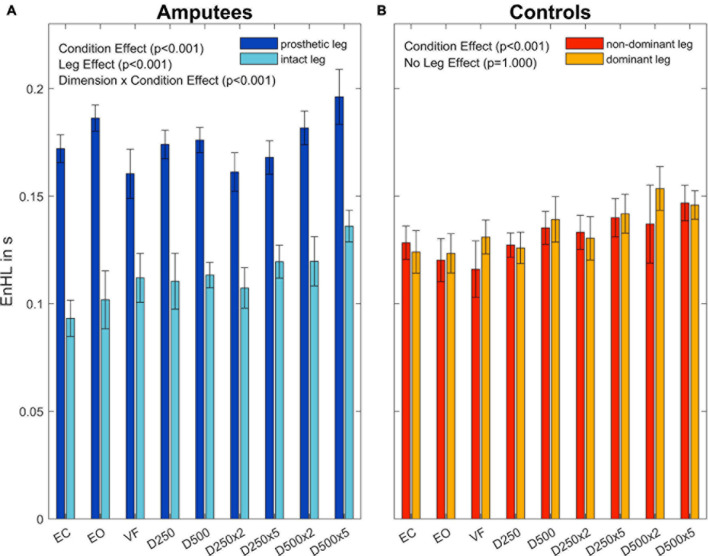
Entropic half-life (EnHL) for the different legs in amputees **(A)** and controls **(B)** for the different augmented visual feedback conditions (VF, visual feedback of CoP position without delay; D250, with a delay of 250 ms; D500, with delay of 500 ms; D250x2, D250x5, D500x2, and D500x5: with delays of 250 and 500 ms combined with a two- or five-fold amplification of the CoP displacements; EC, with eyes closed; and EO, with eyes open without visual feedback of the CoP position). Error bars represent standard errors.

To further investigate the Leg×Group interaction, a pairwise *t*-test with pooled standard deviation was computed between the EnHL values of the intact/dominant leg, the amputated/non-dominant leg, and both legs together (Table 4). There was no significant difference between the groups regarding the EnHL of both legs (p <  1,00, Figure 6). In the group of amputees, the *post-hoc* test showed a significant increase of the EnHL values of the amputated leg compared to the intact leg (p < 0.001, Figure 6). The EnHL values of the intact leg and both legs of the amputees were different (p < 0.001, Figure 6), as well as the EnHL values of both legs and the amputated leg (p < 0.001, Figure 6). There was no significant difference between the legs of controls (Figure 6). The EnHL values of the amputated leg were significantly higher than those of the non-dominant leg in controls (p < 0.001), and the intact leg was significantly lower than the dominant leg in the control group (p < 0.001).

**TABLE 4:** *p*-values from pairwise *t*-test with pooled standard deviation to test for significant difference between the EnHL values of the different Legs for amputees and controls.

	**A**	**Both (A)**	**Both (C)**	**D**	**I**
Both (A)	**<0.001**	–	–	–	–
Both (C)	**<0.001**	1.000	–	–	–
D	**<0.001**	0.059	1.000	–	–
I	**<0.001**	**0.002**	**<0.001**	**<0.001**	–
ND	**<0.001**	0.976	1.000	1.000	**<0.001**

*Both (A), Both Legs, Amputees; I, Intact Leg; A, Amputated Leg; Both (C), Both Legs, Controls; D, Dominant Leg; ND, Non-dominant Leg. Statistically significant values are displayed in bold font (significance level α = 0.05).*

For the group of amputees, the EnHL values of the visual feedback condition D500x5 were significantly greater than all other conditions, except for the D500x2 condition (Table 5). The EnHL values of the EC condition were significantly smaller than the ones from all the visual feedback conditions with a higher delay time and amplification than the D250x5 condition (Table 5). There was a significant difference between the EnHL values of the D500x5 and D250x2 conditions (p < 0.001, Table 5). The EnHL values from the visual feedback condition D500x2 was significantly greater than the VF feedback condition (p =  0.037, Table 5). The EnHL values of the D500x2 and D250x2 visual feedback conditions differed significantly (p < 0.001, Table 5). The EnHL values of the D250x2 differed also significantly from the D250x5 condition (p = 0.041,Table 5).

**TABLE 5:** *p*-values from pairwise *t*-test with Bonferroni-correction to test for significant difference between the EnHL values of the different conditions in Amputees.

	**D250**	**D250x2**	**D250x5**	**D500**	**D500x2**	**D500x5**	**EC**	**EO**
D250x2	1.000	–	–	–	–	–	–	–
D250x5	1.000	**0.041**	–	–	–	–	–	–
D500	1.000	0.334	1.000	–	–	–	–	–
D500x2	0.359	**0.001**	1.000	1.000	–	–	–	–
D500x5	**<0.001**	**<0.001**	**<0.001**	**<0.001**	0.051	–	–	–
EC	0.518	1.000	**0.017**	**0.029**	**<0.001**	**<0.001**	–	–
EO	1.000	1.000	1.000	1.000	1.000	**0.001**	0.142	–
VF	1.000	1.000	0.950	1.000	**0.037**	**<0.001**	1.000	1.000

*Statistically significant values are displayed in bold font (significance levelα = 0.05).*

In the control group, there was a significant increase of the EnHL values in the D500x5 condition, compared to the ones from the EC, EO, VF, D250, and D250x2 conditions (Table 6). There was a significant difference between the EnHL values of the D250x5 condition and the ones of the D250, the EC, EO, and the VF conditions (Table 6). The EnHL values of the D500 condition differed from ones of the D250 (*p* =  0.001, Table 6) and from the EO (*p* =  0.033, Table 6). The EnHL values of the D500 were different from the ones from the VF condition (*p* =  0.021, Table 6). The EnHL values of the D500x2 condition were higher than the ones of the VF condition (*p* =  0.001,Table 6).

**TABLE 6:** *p*-values from pairwise *t*-test with Bonferroni-correction to test for significant difference between the EnHL values of the different conditions in controls.

	**D250**	**D250x2**	**D250x5**	**D500**	**D500x2**	**D500x5**	**EC**	**EO**
D250x2	1.000	–	–	–	–	–	–	–
D250x5	**<0.001**	0.256	–	–	–	–	–	–
D500	**0.001**	1.000	1.000	–	–	–	–	–
D500x2	0.067	1.000	1.000	1.000	–	–	–	–
D500x5	**<0.001**	**0.023**	1.000	1.000	1.000	–	–	–
EC	1.000	1.000	**0.028**	0.346	0.300	**0.002**	–	–
EO	1.000	1.000	**0.003**	**0.033**	0.062	**<0.001**	1.000	–
VF	1.000	1.000	**0.001**	**0.021**	**0.001**	**0.001**	1.000	1.000

*Statistically significant values are displayed in bold font (significance level α = 0.05).*

Since there was an interaction effect between the directions and the visual feedback conditions in the group of amputees (*p* = 0.015, Supplementary Table 5), pairwise *t*-tests with Bonferroni correction for the two directions and the visual feedback conditions were performed. The results showed more significant differences between visual feedback conditions in the ML direction than in the AP direction in the group of amputees (Supplementary Figure 1 and Supplementary Tables 7, 8). No significant differences were observed for the directions in the group of controls (*p* = 0.526, Supplementary Figure 2, and Supplementary Table 6).

## Discussion

In this study, we investigated how unilateral transfemoral amputees incorporate visual feedback of their CoP position during balance control. Receiving real-time visual feedback of the CoP position without delay or amplification did not reduce the sway area compared to the EO condition, neither in the group of amputees nor in controls. Amputees faced more difficulties when incorporating augmented visual feedback to control their CoP dynamics, resulting in a larger sway area than the able-bodied control group. The dynamics of the CoP adjustments of the amputated leg were significantly lower than the intact leg and the dominant and non-dominant legs of controls. With increasing delay and amplification, both groups were able to compensate for small visual disturbances, but their CoP dynamics were significantly lower when additional CoP information was not obtained in a physiological relevant time frame. This was particularly the case with delay times of 500 ms and amplifications of two- and five-folds.

Providing real-time visual feedback of the CoP position without delay or amplification did not result in a reduction of the sway area (Figure 3). Since amputees rely more on their visual system (Barnett et al., 2013; Ku et al., 2014), we predicted a reduction of their sway area during the VF condition. However, this was not the case neither for amputees nor for controls. In fact, the amputee group presented a larger sway area than controls (*p* = 0.046, Figure 3). This group difference disappeared after normalizing the sway area values with respect to the EO condition (Figure 4). Thus, although the sway area was larger in amputees than in controls, disrupted visual feedback of the CoP position (delayed or amplified) produced the same disturbance in the postural control of both groups (i.e., sway area increased with increased delays and amplification). Despite the use of their prosthesis and their incomplete sensory feedback, amputees seem to react similarly to visual feedback of their CoP as controls. Perhaps, maintaining the CoP position within the target circle may have not been intuitive enough and may have induced an attentional shift toward the task goal instead of focusing on standing still, as it has been previously reported in healthy young adults (Kręcisz and Kuczyński, 2018).

The EnHL values of the surrogate CoP data were significantly lower than the ones from the original CoP data, confirming that the CoP adjustments were not the result of random processes. The dynamics of the CoP adjustments of amputees were not significantly different from those of controls when looking at the contributions of both legs. However, there were differences in the dynamics of the CoP adjustments in response to different visual conditions (Group×Condition interaction, *p* =  0.005, Figure 6; and Group×Leg interaction, *p* = < 0.001, Figure 6) when looking at each leg separately. The group of amputees displayed lower dynamics in the amputated leg compared to the intact leg (Figure 6). Compared to the dominant and non-dominant leg of controls, the dynamics of the amputated leg were significantly lower as well. Thus, the somatosensory contributions of the stump were not enough to compensate for the lack of sensory feedback from the absent foot and knee. This was evidenced in the difference in EnHL values between the amputated and the intact limb, and the amputated limb and the dominant and non-dominant limbs of controls (Table 4). Further, the intact leg presented higher dynamics than the dominant leg of the controls. This is in line with our previous work where similar patterns in the dynamics of the CoP adjustments were observed without visual feedback of the CoP position (Claret et al., 2019). Kręcisz and Kuczyński (2018) as well as Lakhani and Mansfield (2015) observed a higher irregularity in the CoP data and increased reaction times when visual feedback of the CoP position was provided in young healthy adults. In our study, the dynamics of CoP adjustments while providing unmodified real-time visual feedback (VF condition) did not differ from the ones during the EO and the EC conditions regardless of the group (Tables 4, 5). However, our work analyzed an older population than the one reported by Krecisz and Kuczynski (2018), and Lakhani and Mansfield (2015).

The loss of sensory feedback may lead to a reduction of the solution space of possible ways in which a movement can be performed, resulting in a less flexible and therefore less adjustable system (Pasluosta et al., 2018; Claret et al., 2019). With less flexibility, the ability to execute precise and quick adjustments may be restricted, leading to increased EnHL values in the amputated leg. Since the amputees may fail to perform fast movements with the prosthetic leg, the intact leg has to produce even more and faster adjustments to compensate for the impairments in the amputated leg. The fast adjustments would explain the lower EnHL values in the intact leg (Federolf et al., 2015; Claret et al., 2019). Further, since there was no group effect in the EnHL values (Table 3), it seems that the intact leg was able to fully compensate for the lower dynamics of the prosthesis side, but failing in reducing the sway area (Group effect, Figure 3). On the other hand, longer EnHL values might be a sign that higher cognitive functions for computing sensory information and postural responses were involved. The processing time by the CNS usually requires up to 100 ms for higher cognitive functions, which is a much longer time than the one required by reflex circuits of lower-level processes typically involved in postural movements (20–50 ms) (Federolf et al., 2015). Further, vision is associated with delays 40–50 ms longer than proprioceptive information (Cameron et al., 2014). Thus, longer EnHL values in the amputated leg may stand for a predominant usage of visual information and higher cognitive functions of postural control. All in all, the posture controllability in amputees was kept in the same range as in controls by increasing the dynamics of the intact limb to compensate for lower dynamics on the amputation side. The brain performed these adaptations without the need of altering the sway area to keep the body in a stable posture. This is a different strategy from the one observed in patients with neurological disorders affecting the CNS (such as Parkinson’s disease), where the resulting dynamics are lower compared to healthy controls as a result of decreased control abilities of the brain (Pasluosta et al., 2018).

We hypothesized that when confronted with delayed and amplified visual feedback of the CoP position, the sway area would increase for both groups, but that amputees would show different CoP dynamics since their postural system is more rigid due to the mechanics of the prosthetic ankle and the disrupted somatosensory capabilities. The results showed a significantly higher sway area between the EO condition and the EC condition, which is in agreement with previous findings under similar visual feedback experiments (Nadollek et al., 2002; Curtze et al., 2012). The EO condition led to a significantly smaller sway area than for the conditions with visual feedback of the CoP position (Table 5). Delayed but not amplified visual feedback of the CoP position resulted in a sway area similar to the EO condition in both groups. Also in both groups, the EC condition resulted in the lowest EnHL values and the D500x5 condition in the highest EnHL values. Lower EnHL values during the EC condition compared to the one obtained during EO were also observed in our previous work (Claret et al., 2019). The lack of visual feedback may have led participants to rely more on somatosensory feedback, which is reflected in shorter processing times (Cameron et al., 2014). As a consequence, this could lead to more adjustments in a shorter time frame resulting in a less precise control (Claret et al., 2019). Contrary, the higher EnHL values during the D500x5 condition may be the result of relying heavily on visual feedback while performing the balance task. With higher delays and amplification, controlling the CoP position becomes increasingly difficult. Since all the participants tended to overcompensate the movements during the conditions with large visual feedback delays, the time between CoP adjustments was also longer. The participants had to wait for the delay time of the CoP to see the actual result of the previous adjustment and to be able to react accordingly. This motion pattern led to an increased sway area and higher EnHL values in both amputees and able-bodied participants. Thus, if visual feedback does not provide information to the CNS in a physiologically meaningful time frame, it will rather destabilize the postural behavior (van den Heuvel et al., 2009).

Our findings should not be considered without accounting for some experimental limitations. First, age-related changes in the CoP dynamics might have yielded higher CoP velocities and CoP sway areas in our rather old study population compared to younger subject groups. Moreover, with the current experimental settings, it is not possible to distinguish whether the observed results derive from neural or mechanical aspects of postural control in amputees. Further studies should focus on experimental paradigms where the distinction between the origin of the CoP dynamics could be ruled out, which may be achieved by including EMG or EEG measurements. More dynamic tasks with more complex visual paradigms could provide further explanations for the results of this study. Even though the experimental settings of this work did not allow to assess rigorously the contributions of the vestibular system, proprioceptive feedback is far more sensitive and faster than the vestibular system to perceive changes in postural sway (Fitzpatrick and McCloskey, 1994). Therefore, we hypothesize little effects in the EnHL associated with alterations in the vestibular system. However, this hypothesis needs to be proved with further experimentation. Future work should also focus on how restoring sensory feedback in this patient population *via* surface or intraneural electrical stimulation may improve their performance in such balance tasks.

## Data Availability Statement

Data supporting the conclusions of this article will be available by the corresponding author upon request.

## Ethics Statement

The studies involving human participants were reviewed and approved by Ethics Committee of the University of Freiburg (ethical approval N° 230/18). The patients/participants provided their written informed consent to participate in this study.

## Author Contributions

KF performed the data collection, data processing, statistical analysis, and analyzed the results. TK, TL, and LK helped with the data collection and analysis of the results. MM helped to recruit the participants. CP designed the study protocol and helped to analyze, and to interpret the results. GH, VV, NS, and TS helped in interpreting the results. VV developed the EnHL measure. All the authors helped to prepare the manuscript.

## Conflict of Interest

The authors declare that the research was conducted in the absence of any commercial or financial relationships that could be construed as a potential conflict of interest.

## Publisher’s Note

All claims expressed in this article are solely those of the authors and do not necessarily represent those of their affiliated organizations, or those of the publisher, the editors and the reviewers. Any product that may be evaluated in this article, or claim that may be made by its manufacturer, is not guaranteed or endorsed by the publisher.

## Abbreviations

AP: anterior-posterior
BoS: base of support
CNS: central nervous system
COM: center of mass
CoP: center of pressure
D250: delayed by 250 ms
D250x2, delay time 250 ms: twofold amplification
D250x5, delay time 250 ms: fivefold amplification
D500: delayed by 500 ms
D500x2, delay time 500 ms: twofold amplification
D500x5, delay time 500 ms: fivefold amplification
EC: eyes closed
EO: eyes open
ML: mediolateral
VF: visual feedback.
