# Supplementary material for: Influence of Augmented Visual Feedback on Balance Control in Unilateral Transfemoral Amputees
Source: Front Neurosci. 2021 Sep 13;15:727527. doi: 10.3389/fnins.2021.727527 (PMC8473899; doi:10.3389/fnins.2021.727527)
Supplement: Supplementary file 1 [file Data_Sheet_1.pdf]

Influence of augmented visual feedback on balance control in unilateral transfemoral amputees

K. Fuchs[1], T. Krauskopf[1], T. B. Lauck[1], L. Klein[2], G. W. Herget[2], V. Von Tscharner[3], N. Stutzig[4], T. Stieglitz[1,5,6]†, C. Pasluosta[1]†*

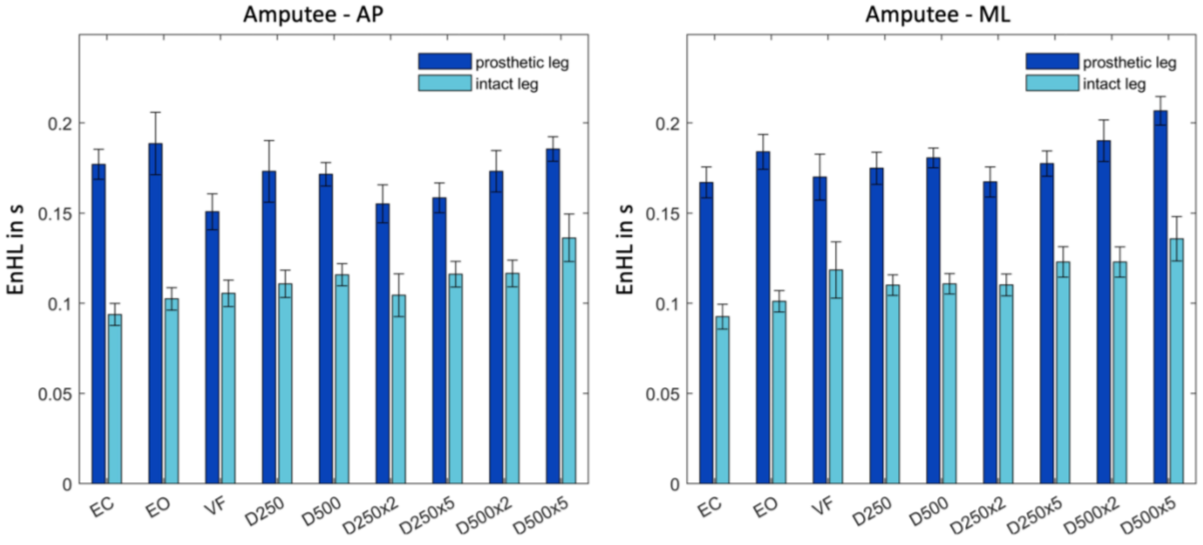

S 1: EnHL of the amputated leg compared to the intact leg in anterior-posterior (left) and medio-lateral directions (right).

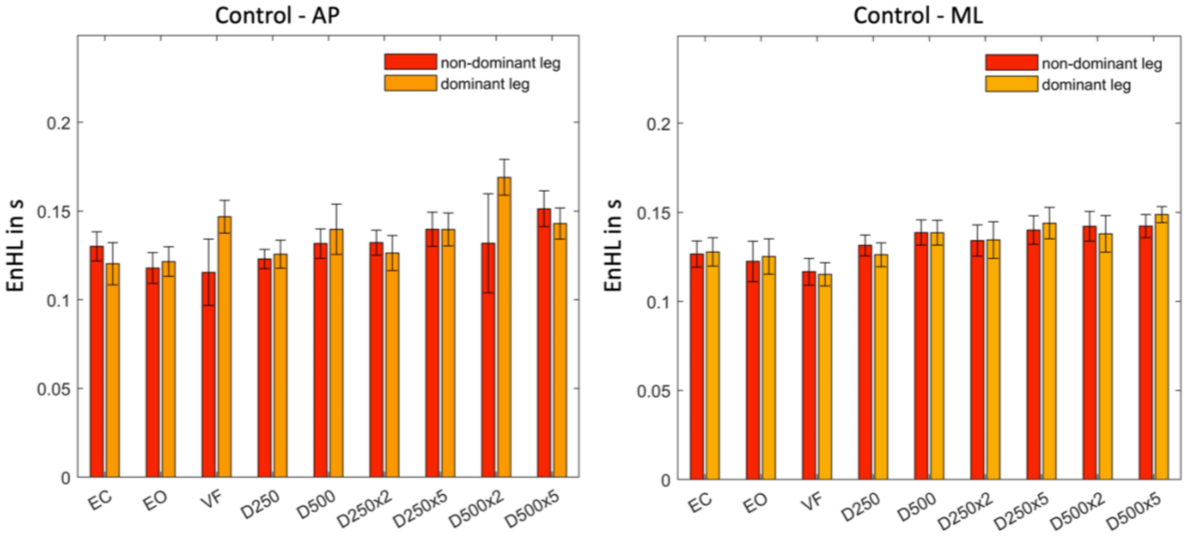

S 2: EnHL of the non-dominant leg compared to the dominant leg in anterior-posterior and medio-lateral directions.

Supplementary Table 1: Two-way mixed ANOVA of the sway area of the amputees and controls. Statistically significant values are displayed in bold font and marked with an asterisk (significance level α = 0.05, p-value: * p ≤ 0.05, ** p ≤ 0.01, *** p ≤ 0.001).

Main- and Interaction Effects	F-value	p<.05	p-value
**Group**	**4.59**	*	**0.046**
**Condition**	**9.36**	***	**<0.001**
Group x Condition	2.69		0.109

Supplementary Table 2: Two-way mixed ANOVA of the sway area ratio data normalized to the eyes open condition of the amputees and controls. Statistically significant values are displayed in bold font and marked with an asterisk (significance level α = 0.05, p-value: * p ≤ 0.05, ** p ≤ 0.01, *** p ≤ 0.001).

Main- and Interaction Effects	F-value	p<.05	p-value
Group	0.54		0.473
**Condition**	**7.89**	***	**<0.001**
Group x Condition	1.59		0.131

Supplementary Table 3: Pairwise t-test with Bonferroni-correction to test for significant difference between the sway area ratio data normalized to the eyes open condition in amputees and controls. Statistically significant values are displayed in bold font (significance level α = 0.05).

	D250	D250x2	D250x5	D500	D500x2	D500x5	EC	EO
**D250x2**	0.607	-	-	-	-	-	-	-
**D250x5**	1.000	1.000	-	-	-	-	-	-
**D500**	1.000	0.141	1.000	-	-	-	-	-
**D500x2**	0.426	1.000	1.000	0.557	-	-	-	-
**D500x5**	0.095	0.854	1.000	0.076	1.000	-	-	-
**EC**	**0.002**	**0.016**	1.000	**0.001**	0.501	1.000	-	-
**EO**	0.153	**0.003**	0.695	0.050	**0.032**	**0.009**	**< 0.001**	-
**VF**	1.000	1.000	1.000	1.000	1.000	0.501	**0.002**	0.084

Supplementary Table 4: Five-way mixed ANOVA of the EnHL data. Statistically significant values are displayed in bold font and marked with an asterisk (significance level α = 0.05, p-value: * p ≤ 0.05, ** p ≤ 0.01, *** p ≤ 0.001). Verification of the significant difference between original and surrogate data (signal), to confirm the non-random origin of the EnHL-values of the original signal.

Main- and Interaction Effects	F-value	p<.05	p-value
**Signal**	**146.906**	***	**<0.001**
**Group**	**7.624**	**	**0.006**
Direction	0.229		0.633
**Leg**	**50.773**	***	**<0.001**
**Condition**	**47.380**	***	**<0.001**
Signal x Group	0.228		0.633
Signal x Direction	0.104		0.747
Group x Direction	0.377		0.540
Signal x Leg	0.769		0.465
**Group x Leg**	**56.865**	***	**<0.001**
Direction x Leg	1.142		0.321
Signal x Condition	1.005		0.424
**Group x Condition**	**5.539**	***	**<0.001**
Direction x Condition	1.230		0.285
**Leg x Condition**	**1.707**	*	**0.039**
Signal x Group x Direction	0.458		0.499
Signal x Group x Leg	0.672		0.512
Signal x Direction x Leg	0.438		0.646
Group x Direction x Leg	0.057		0.945
Signal x Group x Condition	0.561		0.780
Signal x Direction x Condition	0.865		0.530
**Group x Direction x Condition**	**2.065**	*	**0.047**
Signal x Leg x Condition	0.784		0.681
Group x Leg x Condition	1.533		0.096
Direction x Leg x Condition	0.788		0.678
Signal x Group x Direction x Leg	0.084		0.919
Signal x Group x Direction x Condition	1.342		0.218
Signal x Group x Leg x Condition	0.684		0.785
Signal x Direction x Leg x Condition	0.654		0.813

Group x Direction x Leg x Condition	0.838	0.622
Signal x Group x Direction x Leg x Condition	0.237	0.998

Supplementary Table 5: Three-way mixed ANOVA of the original EnHL-values of the Amputees. to test for significant differences between the leg. Direction and condition. Statistically significant values are displayed in bold font and marked with an asterisk (significance level α = 0.05, p-value: * p ≤ 0.05, ** p ≤ 0.01, *** p ≤ 0.001).

Main- and Interaction Effects	F-value	p<.05	p-value
Direction	0.735		0.395
**Leg**	**42.431**	***	**<0.001**
**Condition**	**13.67**	***	**<0.001**
Direction x Leg	0.142		0.868
**Direction x Condition**	**2.686**	*	**0.015**
Leg x Condition	1.642		0.080
Direction x Leg x Condition	0.353		0.977

Supplementary Table 6: Three-way mixed ANOVA of the original EnHL-values of the Controls to test for significant differences between the leg Direction and condition. Statistically significant values are displayed in bold font and marked with an asterisk (significance level α = 0.05, p-value: * p ≤ 0.05, ** p ≤ 0.01, *** p ≤ 0.001).

Main- and Interaction Effects	F-value	p<.05	p-value
Direction	0.045		0.832
Leg	0.280		0.757
**Condition**	**8.833**	***	**<0.001**
Direction x Leg	0.112		0.895
Direction x Condition	0.833		0.526
Leg x Condition	0.589		0.820
Direction x Leg x Condition	1.025		0.422

Supplementary Table 7: Pairwise t-test with Bonferroni-correction to test for significant difference between the EnHL-data of the different conditions in AP direction in amputees. Statistically significant values are displayed in bold font (significance level α=0.05).

AP	D250	D250x2	D250x5	D500	D500x2	D500x5	EC	EO
**D250x2**	1.000	-	-	-	-	-	-	-
**D250x5**	1.000	1.000	-	-	-	-	-	-
**D500**	1.000	0.737	1.000	-	-	-	-	-
**D500x2**	1.000	0.191	1.000	1.000	-	-	-	-
**D500x5**	0.402	**<0.001**	**0.002**	0.079	0.450	-	-	-
**EC**	1.000	1.000	1.000	1.000	1.000	**0.009**	-	-
**EO**	1.000	0.682	1.000	1.000	1.000	1.000	1.000	-
**VF**	0.709	1.000	1.000	**0.033**	0.149	**<0.001**	1.000	0.211

Supplementary Table 8: Pairwise t-test with Bonferroni-correction to test for significant difference between the EnHL-data of the different conditions in ML direction in amputees. Statistically significant values are displayed in bold font (significance level α=0.05).

ML	D250	D250x2	D250x5	D500	D500x2	D500x5	EC	EO
**D250x2**	1.000	-	-	-	-	-	-	-
**D250x5**	1.000	0.117	-	-	-	-	-	-
**D500**	1.000	1.000	1.000	-	-	-	-	-
**D500x2**	0.076	**0.039**	1.000	1.000	-	-	-	-
**D500x5**	**0.001**	**<0.001**	**0.026**	**<0.001**	1.000	-	-	-
**EC**	0.507	0.451	**<0.001**	**0.038**	**0.001**	**<0.001**	-	-
**EO**	1.000	1.000	1.000	1.000	0.234	**0.001**	0.982	-
**VF**	1.000	1.000	1.000	1.000	1.000	**0.004**	0.969	1.000
